# Urban and rural differences in needs, service use and satisfaction among caregivers of autistic children in Morocco

**DOI:** 10.1177/13623613221150086

**Published:** 2023-02-17

**Authors:** Maretha de Jonge, Mohamed Boutjdir, Tahar El-Korchi, Hafida Torres, Arun Karpur, Andy Shih, Abdeslem Elidrissi

**Affiliations:** 1 Leiden University, The Netherlands; 2 VA New York Harbor Healthcare System, USA; 3 State University of New York Downstate Health Sciences University, USA; 4 NYU Grossman School of Medicine, USA; 5 Worcester Polytechnic Institute, USA; 6 American Moroccan Competencies Network, USA; 7 Autism Speaks, Inc., USA; 8 College of Staten Island, USA

**Keywords:** autism, caregivers, low-resource area, Morocco, rural

## Abstract

Understanding caregivers’ needs is crucial for service planning and empowering caregivers around the world. Although research on autism from Arab countries is emerging, many countries are still unrepresented. Moreover, within-country disparities are understudied. This study investigated differences in needs, service use, and satisfaction between urban or rural living caregivers of autistic children in Morocco. The “Andersen Behavioral Model of Health Services Use” served as a conceptual framework to investigate factors associated with receiving intervention and service satisfaction. Results from a survey among 131 Moroccan caregivers of autistic children revealed similarities and differences in urban and rural living caregivers’ challenges and needs. Children from urban communities had 12 times higher odds of receiving intervention despite similar age and verbal ability. Significantly, fewer children from rural communities attended school as compared with urban communities. Limited autonomy skills in children were more challenging to rural caregivers, while limited social-communicational skills were more challenging to urban caregivers. These differences may inform healthcare policy-makers and program developers. Adaptive interventions are important to reflect regional needs, resources, and practices. Addressing enabling factors such as costs, information barriers, or stigma may help reduce both global and within-country disparities in autism care.

**Lay Abstract:**

It is very important to understand the needs of caregivers to be able to empower caregivers and to develop or improve services around the world. Therefore, research in different regions is needed to understand differences in caregivers needs between countries, but also between areas within countries. This study investigated differences in needs and service use between caregivers of autistic children in Morocco, living in urban and rural areas. A total of 131 Moroccan caregivers of autistic children took part in the study and responded to an interview survey. The results showed both similarities and differences between urban and rural living caregivers’ challenges and needs. Autistic children from urban communities were much more likely to receive intervention and attend school than children from rural communities, even though age and verbal skills of the two groups of children were comparable. Caregivers expressed similar needs for improved care and education, but different challenges in caring. Limited autonomy skills in children were more challenging to rural caregivers, while limited social-communicational skills were more challenging to urban caregivers. These differences may inform healthcare policy-makers and program developers. Adaptive interventions are important to respond to regional needs, resources, and practices. In addition, the results showed the importance of addressing challenges as experienced by caregivers such as costs related to care, barriers in access to information, or stigma. Addressing these issues may help reduce both global and within-country differences in autism care.

## Introduction

The needs of caregivers of autistic children, to understand their children’s development, to seek and receive adequate support, and to be included in the society, are universal worldwide. Still, there are significant global inequalities in resources and access to services (Lord et al., 2022).^1^ Over the last decade, studies on the prevalence and the impact of autism in low- and middle-income countries (LMICs) have increased (Zeidan et al., 2022). Studies from both LMICs and high-income countries (HICs) have shown that caring for autistic children often places heavy strains on caregivers. Caregivers report high levels of stress, anxiety, and depression. They continually prioritize the needs of their autistic child, over the needs of other family members or their own needs, and struggle with the burden of time management, work, and financial constrictions (Bonis, 2016; Boshoff et al., 2016; Gona et al., 2016). They report support, and also frequent negative reactions from extended family members, as well as stigma, isolation, and frustration with access to services or education (Landon et al., 2018; Ooi et al., 2016; Singh et al., 2017). Burden on family resources and functioning was found to be higher for caregivers of autistic children as compared to children with other developmental disabilities (Rogge & Janssen, 2019; Vohra et al., 2014; Wang et al., 2011).

Despite global similarities in experiences, there are important regional or cultural differences between caregivers’ perceptions, needs and challenges, and within and between regions (de Leeuw et al., 2020). It is therefore important to investigate caregivers’ experiences in specific regions. In the Arab Middle Eastern and North African (MENA) countries, research on autism is emerging (Alnemary et al., 2017). A recent review revealed significant psychological distress in caregivers in MENA countries (Alallawi et al., 2020). Stigma or lack of social support, also within extended families that play an important role in MENA societies, is often mentioned (Alkhateeb et al., 2016; Lamba et al., 2022; Obeid et al., 2015; Shattnawi et al., 2021). Limited knowledge about autism and limited access to information, due to language barriers or lack of services, contributed to caregivers stress (AlKhateeb et al., 2019; Zaki & Moawad, 2016). Limited access to services affects not only the caregivers, but also the development opportunities for children. For example, Palestinian caregivers indicated that their autistic children were often excluded from schools (Dababnah & Bulson, 2015). In Qatar, young autistic children spent more time indoors watching television than children without autism, which was explained as a sign of possible helplessness of parents, or as a result of stigma and lack of services (Kheir et al., 2012). The MENA region is a large region and despite religious and cultural similarities, there are also important differences between countries with respect to national income, health care organization, geographic factors, and urbanization (Asbu et al., 2017; McKee et al., 2017). The Eastern part of the region includes HIC Gulf countries, and so far, most studies on autism are conducted in this part. There is a lack of studies from the Western part, including Morocco (Alallawi et al., 2020).

Caregivers’ burden was also found to be affected by within-region factors such as rural or urban living areas, due to geographic barriers, limited autism resources, low educational attainment, or cultural perspectives (Antezana et al., 2017; Ault et al., 2021; Jain et al., 2019). However, few studies made comparisons between urban and rural caregivers, and information regarding caregiver needs from rural areas is limited (Ault et al., 2021). To support caregivers of autistic children, it is crucial to investigate their challenges, needs, and service utilization on a regional level. For the development of effective services and responsive mental health care programs, within-regional differences between rural or urban living families should be carefully considered.

This study investigated the needs of caregivers of autistic children in Morocco. The revisited “Andersen Behavioral Model of Health Services Use” (Andersen, 1995) served as a conceptual framework to investigate factors associated with receiving pertinent services for children, and with caregivers’ service satisfaction. Andersen’s model has widely been used (Babitsch et al., 2012) and posits that service utilization can be explained by a combination of predisposing factors to use services (e.g. socio-demographic factors), enabling factors that facilitate access to care and need factors (e.g. severity of disability). In turn, this may affect the evaluated health status and consumer satisfaction (Andersen, 1995). A recent study in the United States showed that predisposing factors and need factors predicted unmet health care needs among autistic children, while adding the enabling factors (i.e. health insurance, financial means) improved the prediction significantly (Karpur et al., 2019). Among Australian caregivers of autistic children, predisposing factors (i.e. higher parental education), and enabling factors (i.e. financial means) predicted service use and satisfaction. The need-based factors were identified as the strongest predictors of service use (Willet et al., 2018).

This study is first to investigate difference in needs and challenges between caregivers living in urban and rural areas in Morocco. Understanding regional similarities and differences in needs, service usage, and service satisfaction may help empowering caregivers and may support policy-makers to implement services that will be beneficial in Morocco and other similar urban and rural communities in MENA regions. Therefore, the aims of this study are to (1) document challenges and needs of caregivers, (2) investigate differences in caregivers’ needs and service use in urban and rural areas of Morocco, and (3) explore predisposing, enabling, and need factors associated with service use and satisfaction, as perceived by caregivers, informed by Andersen’s (1995) model.

## Methods

A cross-sectional survey study was conducted among caregivers of autistic children. Using convenience sampling, participants were recruited through parent associations, nongovernmental organizations (NGOs) for children with developmental disorders and special schools.

### Measures

**Figure 1.**
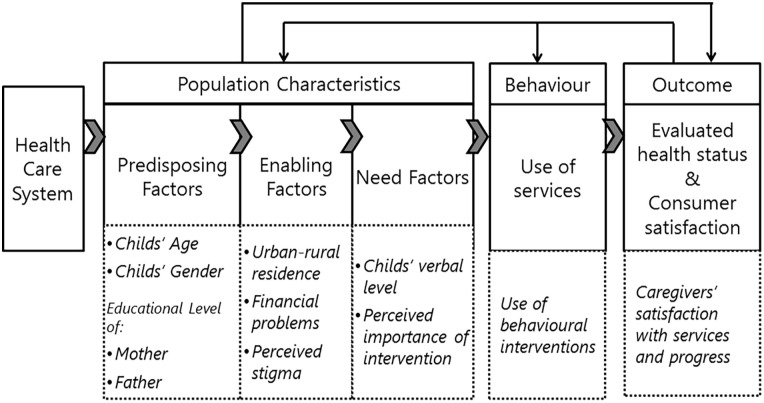
Application of Andersen’s model to predict service use and satisfaction.

The Caregiver Needs Survey developed by Autism Speaks and previously used in Southeast Europe was utilized to collect the data (Daniels et al., 2017). The survey comprises four domains: respondent characteristics, child characteristics, service encounters, and caregiver needs and perceptions. The variables representing predisposing, enabling, and need factors in our study, as well as service usage and satisfaction, are presented in Figure 1.

The *predisposing factors* comprised child’s age, gender, fathers’, and mothers’ educational levels. The enabling factor included urban or rural residency, financial problems (one item) and stigma. Four items from the affective dimension of the Affiliate Stigma Scale (Mak & Cheung, 2008) were incorporated in the Caregiver Needs Survey. The items are scored on a 4-point Likert-type scale (see Supplementary Table). Cronbach’s alpha of the Stigma Scale in our sample was 0.63 (no item subscale correlations below 0.30). *The needs factor* comprised the child’s verbal level coded on a 5-point scale (ranging from 1 = “no speech” to 5 = “uses complex or compound sentences”), and the perceived level of importance of autism support services. Importance of support services and services satisfaction, as perceived by caregivers was measured by the Support Subscale of the Family Quality of Life Survey (FQLS) (Hoffman et al., 2006). Caregivers rated how important it was to them that their children received support at home, at school, in situations with peers and at service-provider settings (FQLS—importance subscale, four items). Subsequently, they rated their satisfaction with the support services in each of these settings (FQLS—satisfaction, four items). The items are coded on 3-point Likert-type scales (see Supplementary Table). Cronbach’s alphas in our sample were FQLS—importance = 0.62 and FQLS—satisfaction = 0.75 (no item-subscale correlations below 0.30). See the work of Daniels et al. (2017) for an extensive description of the Caregiver Needs Survey.

Child intervention service use was investigated by asking caregivers to select which interventions they received at past and present. Pharmacological interventions were separated from non-pharmacological interventions targeting skills or behavior (e.g. speech–language therapy, applied behavior analysis (ABA), psycho-motor therapy). Multiple choice selection questions were used to investigate caregivers’ top 3 challenges in caring and priorities for support. Their top 3 training needs were examined using an open question.

The survey was translated into Arabic and back-translated into English to ensure consistency. Subsequently, it was piloted among a small group of parents and community practitioners from urban and rural communities. Based on their feedback, the wording of several questions was simplified. Some items were adapted slightly to reflect the Moroccan educational system and cultural context (e.g. examples of verbal ability were adapted to the Moroccan context for instance requesting “bread” instead of “bubbles”; “travel distance” was changed into travel time, to better represent travel strains in mountainous areas).

### Data collection

Participants were recruited through parent associations and special schools and invited for the interview. Most respondents (91%) could be interviewed in-person. Eleven caregivers (9%) were not able to participate in the interviews due to time or travel constraints and were offered an online survey. To ensure data accuracy, we checked the completeness of the online surveys. The online respondents all completed the questionnaire and answered to the few open-ended questions. The online respondents completed at least secondary education. We could not detect any differences in data quality between the online and interview data, nor in age of the child, total stigma score, years since diagnosis or financial problems in caring for the child.

The interviews were conducted by two teams of four undergraduate students each. Both teams traveled to centers in urban and in rural communities to interview caregivers in person. The students took part in an academic global studies program of the Worcester Polytechnic Institute and College of Staten Island (CUNY), USA. Three of eight students were native Arabic speakers. The non-Arabic-speaking students were paired up with Moroccan translators. Interviewers and translators were trained to ensure reliable interviews and to avoid bias. They practiced in role-plays before starting data collection. University professors with expertise in global social studies, and in guiding students during exchange projects monitored the training and data collection.

Ethical approval for the study was obtained from the Institutional Review Board of University B. Authorization was obtained from the local authorities of the Ministry of Interior and from the collaborating centers. All participants were informed about the purpose of the study and informed consent was obtained prior to data collection.

### Participants

A total of 194 caregivers completed the survey. Caregivers of adults (*n* = 13), children without a formal diagnosis (*n* = 29), or diagnoses other than autism (*n* = 21) were excluded from this study, resulting in a final sample of 131 caregivers.

The respondents belonged to 5 of the 12 regions of Morocco: 41% lived in urban communities (>100,000 inhabitants) and 59% lived in rural communities (towns or villages with <100,000 inhabitants) (Table 1).

**Table 1.** Predisposing, enabling, and need factors in urban and rural communities.

	Urban		Rural		Total sample		Chi-square (χ^2^) or Mann–Whitney *U*	*p* value
	41% (*n* = 54)		59% (*n* = 77)		(*n* = 131)			
	%	*n*	%	*n*	%	*n*		
**Respondents**								
*Region*								
Casablanca-Settat	33%	18	0%	0	14%	18		
Drâa-Tafilalet	0%	0	35%	27	21%	27		
Fès-Meknès	13%	7	0%	0	5%	7		
Souss-Massa	52%	28	65%	50	60%	78		
Tanger-Tetouan-AlHoceima	2%	1	0%	0	1%	1		
*Relationship to child*								
Mother	67%	36	45%	35	54%	71	χ^2^ = 7.779	*0.02* *
Father	28%	15	52%	40	42%	55		
Other	6%	3	3%	2	4%	5		
**Predisposing factors: caregivers characteristics**								
*Mothers’ educational level*^a^							*U* = 1066	*<0.01* **
No schooling	8%	4	38%	28	26%	32		
Primary school	19%	9	22%	16	20%	25		
Secondary school	25%	12	9%	7	16%	19		
Vocational training	15%	7	19%	14	17%	21		
College/university	25%	12	12%	9	17%	21		
Post-college/university degree	8%	4	0%	0	3%	4		
*Fathers’ educational level*^b^							*U* = 1443	*0.095*
No schooling	10%	5	21%	15	17%	20		
Primary school	19%	9	23%	17	21%	26		
Secondary school	6%	3	10%	7	8%	10		
Vocational training	25%	12	16%	12	20%	24		
College/university	31%	15	22%	16	26%	31		
Post-college/university degree	8%	4	8%	6	8%	10		
**Predisposing factors: child characteristics**								
*Age (median; range)*	6.8	3–16	7.8	2–18	7.3	2–18	*U* = 2304	*0.29*
*Gender (male/female)*^c^	76/23%	41/12	78/22%	60/17	77/22%	101/29	χ^2^ = 0.006	*0.94*
**Enabling factors**								
*Financial problems*^d^	83%	45	71%	53	76%	98	χ^2^ = 2.759	*0.10*
*Stopped/reduced working hours*	57%	31	43%	33	49%	64	χ^2^ = 2.689	*0.10*
*Stigma Scale (median)*	2.5		2.5		2.5		*U* = 2105	*0.90*
**Need factors**								
*Child’s verbal ability*^e^							χ^2^ = 2.712	*0.10*
No words	31%	17	42%	32	37%	49		
Single words	35%	19	36%	28	36%	47		
Two- to three-word phrases	13%	7	10%	8	11%	15		
Short sentences	11%	6	4%	3	7%	9		
Complex sentences	9%	5	6%	5	8%	10		
*FQLS*—*importance scale (median)*	2.8		3.0		3.0		*U* = 2676	0.002**

Missing values: ^a^*n* = 9; ^b^*n* = 10; ^c^*n* = 1; ^d^*n* = 2; ^e^*n* = 1.

See Supplementary Table for Stigma Scale, and FQLS—scale items scores.

* *p*< 0.05; **p< 0.01.

### Data analysis

First, descriptive data are presented for the variables related to the study aims. Differences between caregivers from urban or rural communities were investigated using Chi-square tests for categorical data and Mann–Whitney *U* tests for ordinal variables (SPSS version 25).

Second, factors associated with service use for obtaining diagnosis and supports were explored. A binominal logistic regression analyses was conducted to examine the combination of predisposing, enabling, and need factors that best predicted the likelihood of receiving non-pharmacological child intervention. Based on the requirement of at least 10 cases per predictor for logistic regression analyses (Vittinghoff & McCulloch, 2007), our sample size was assumed to be adequate. Third, we used a hierarchical linear regression analysis to explore the extent to which the predisposing, enabling, and need factors, and the use of any non-pharmacological child intervention, predicted the FQLS—satisfaction mean score best.

In both regression analyses, predisposing, enabling, and need factors’ variables were entered successively in accordance with Andersen’s model. Results were interpreted as significant when *p* < 0.05.

#### Community involvement statement

Parents of autistic children and community practitioners from urban and rural communities in Morocco were involved in piloting the survey and in adapting it to the cultural context. Community practitioners and parent associations were involved in, or advised on addressing or reaching out to caregivers. The results will be disseminated by our community partners to caregivers, practitioners and policy-makers.

## Results

Table 1 presents the characteristics of the respondents, and the predisposing, enabling, and need factors of the families affected with autism.

### Predisposing factors: caregiver and child characteristics

In urban communities, mothers more often responded to the survey (67% mothers, 28% fathers) than in rural communities (45% mothers, 52% fathers) (χ^2^(2) = 7.779, *p* = 0.02). In 4% of the cases, the responding caregiver was a family member (aunt and grandmother). Caregivers in urban communities had a higher median level of education in comparison with rural communities, which reached significance for mothers (*U* = 1.066, *z* = –3.799, *p* < 0.001) but not for fathers (*U* = 1.443, *z* = –1.668, *p* = 0.10).

There were no significant differences in age or gender between the children from urban and rural communities (see Table1). The median time since diagnosis was 4 years at the time of the study.

### Enabling factors

Caregivers from rural communities traveled longer to obtain diagnostic services (χ^2^(3) = 7.903, *p* < 0.05), although one-third of the urban caregivers also traveled for more than 2 h.Financial problems in caring for the child were frequently reported, especially in urban communities, although the difference between groups was not significant. Caregivers often reported that a family member had to stop working or reduce hours to care for the child.

Responses to the Stigma Scale showed that feelings of helplessness, discrimination, or negative impact were frequently reported by caregivers in both communities. Still, 66% (strongly) disagreed when asked if they worried about other people knowing about their autistic child.

### Need factors, expressed challenges, and priorities

The level of verbal functioning was limited in most children (73% no speech or single words), with no significant differences between urban and rural communities (*U* = 1.725, *z* = –1.629, *p* = 0.10). Most caregivers expressed an urge for their children to make developmental progress, especially in rural communities (FQLS—importance scale *U* = 2.676, *z* = 3.109, *p* < 0.01).

**Figure 2.**
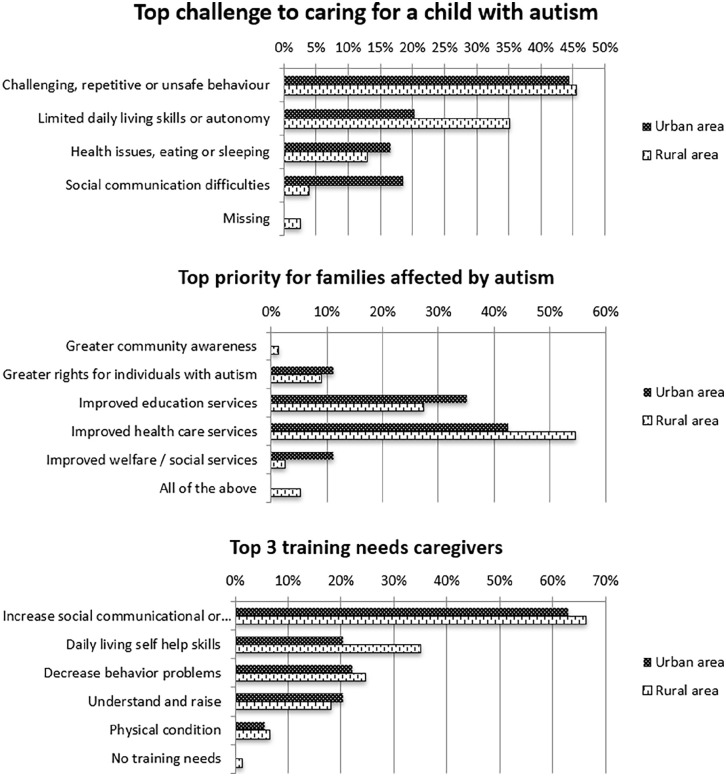
Top challenges, priorities, and training needs of caregivers. Top challenges and priorities were investigated using multiple choice selection questions. Caregivers selected their top 3 challenges and priorities. Caregivers training needs were examined using an open question. The responses were categorized into five themes.

Challenges expressed by caregivers were mostly related to behavior difficulties of the child, followed by limited autonomy skills (i.e. toileting, dressing). The latter was more frequently reported in rural communities (35% vs 20% in urban communities), while social-communicational difficulties were more often a top challenge for urban caregivers (19% vs 4% in rural homes). The above-mentioned differences in types of challenges experienced by urban and rural living caregivers reached significance (χ^2^(4) = 10.908; *p* < 0.05) (Figure 2).

Top priorities for caregivers were improved health care services and improved education for autistic children. Most caregivers requested training to help increase social, communicational, or intellectual skills of their children, followed by training to help improve autonomy skills (30%), decrease behavioral problems (24%), increase parenting skills, and knowledge on autism (19%). Urban and rural caregivers did not significantly differ in their top priorities or training needs (Figure 2).

### Services and support

Diagnostic services were often provided by pediatricians, especially to children in rural communities (53% vs 33% in urban communities) (see Table 2). In urban areas, psychiatrists or psychologists more often assigned diagnoses (respectively, 19, 20% of the cases) than in rural areas (respectively, 8, 13%), but these differences did not reach significance. Overall, about 3% of children received diagnosis from a multidisciplinary team.

**Table 2.** Service use for obtaining diagnoses, intervention, or support.

	Urban		Rural		Total sample		Chi-square (χ^2^) or Mann–Whitney *U*	*p* value
	41% (*n* = 54)		59% (*n* = 77)		*n* = 131			
	%	*n*	%	*n*	%	*n*		
**Obtaining diagnosis**								
*Person first concerned*								
Parent	78%	42	70%	54	73%	96		
Family member	15%	8	23%	18	20%	26		
Health care professional	4%	2	5%	4	5%	6		
Teacher	2%	1	1%	1	2%	2		
Other	2%	1	0%	0	1%	1		
*Travel time to obtain diagnosis*							χ^2^ = 7.903	0.048*
<30 min	54%	29	31%	24	40%	53		
0.5–2 h	17%	9	26%	20	22%	29		
>2 h	30%	16	38%	29	34%	45		
Traveled outside the country	0%	0	4%	3	2%	3		
*Diagnosis given by*							χ^2^ = 7.745	*0.26*
Multidisciplinary team	4%	2	3%	2	3%	4		
Psychiatrist	19%	10	8%	6	12%	16		
Psychologist	20%	11	13%	10	16%	21		
Neurologist	11%	6	13%	10	12%	16		
Pediatrician	33%	18	53%	41	45%	59		
Primary care doctor	4%	2	6%	5	5%	7		
Other/unknown	10%	5	4%	3	7%	8		
*Years since diagnosis (median; range)*^a^	0–15	3.8	0–15	4.1	0–15	4.0	*U* = 1931	*0.72*
**Non-pharmacological interventions for autism**								
*Interventions received in the past*								
Speech–language therapy	57%	31	38%	29	46%	60		
Behavioral intervention (e.g. ABA)	48%	26	26%	20	35%	46		
Social skills training	30%	16	27%	21	28%	37		
Psycho-motor therapy	20%	11	26%	20	24%	31		
Sensory-integration therapy	22%	12	18%	14	20%	26		
Cognitive therapy	20%	11	18%	14	19%	25		
Occupational therapy	15%	8	19%	15	18%	23		
*Interventions received at present*								
Speech–language therapy	48%	26	30%	23	37%	49		
Behavioral intervention (e.g. ABA)	39%	21	23%	18	30%	39		
Social skills training	24%	13	23%	18	24%	31		
Psycho-motor therapy	15%	8	21%	16	18%	24		
Sensory-integration therapy	26%	14	12%	9	18%	23		
Cognitive therapy	19%	10	16%	12	17%	22		
Occupational therapy	13%	7	13%	10	13%	17		
Other	2%	1	3%	2	2%	3		
*No non-pharmacological intervention received*								
Past	15%	8	42%	32	31%	40		
At present	28%	15	51%	39	41%	54		
Past AND present	9%	5	40%	31	27%	36	χ^2^ = 15.306	*<* 0.001***
**Other interventions**								
*Pharmacotherapy (past AND present)*	23%	12	36%	28	31%	40	χ^2^ = 2.561	*0.11*
**Enrolled in school** ^b,c^							χ^2^ = 16.616	*<* 0.01**
Not in school	10%	5	30%	22	23%	27		
Special school	56%	28	21%	15	36%	43		
Special-needs class	16%	8	21%	15	19%	23		
Regular private/public school	18%	9	23%	17	22%	26		
**Support for caregivers**								
*Parent training*^d^	15%	8	13%	10	14%	18		
*Government assistance*^e^	7%	4	4%	3	5%	7		
*Family support group*^f^	24%	13	19%	15	21%	28		

ABA: applied behavior analysis.

Missing values: ^a^*n* = 7; ^b^*n* = 3; ^c^children aged 4–16 years included: *n* = 119; ^d^*n* = 7; ^e^*n* = 4; ^f^*n* = 1.

* *p* < 0.05; **p < 0.01; ***p < 0.001.

**Figure 3.**
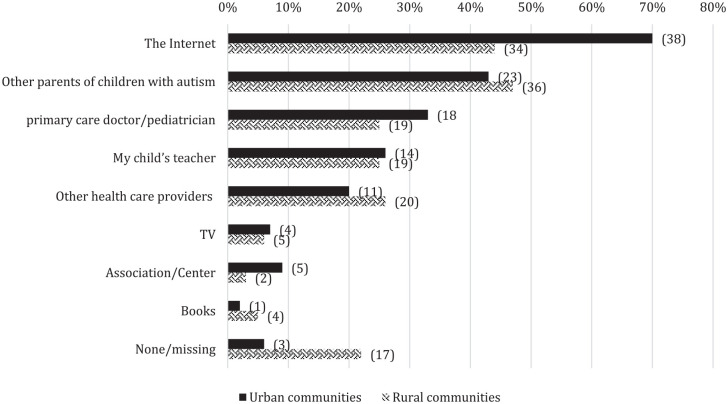
Information sources used by caregivers. Percentage on *x*-axis; *n* between parentheses.

Caregivers’ support comprised family support groups, or parent training, mostly provided by ABA trainers or parent associations. Five percent received government assistance in the form of discounts in public transport or free hospital services. The Internet was reported as an important source of information, although more frequently used by caregivers in urban than in rural communities (70% vs 44%) (Figure 3).

Twenty-two percent of the autistic children aged 4–16 were enrolled in regular education (18% urban and 23% rural). Most children did not receive any in-school special need supports, except for three (urban: *n* = 2 and rural: *n* = 1). In urban communities, many children attended a center for students with disabilities (56%) or a special needs classroom in a regular school (16%) while 10% did not attend school. In rural communities, significantly more children (30%) were not enrolled in school (χ^2^(3) = 16.616, *p* < 0.01).

Non-pharmacological intervention usage differed between urban and rural living families. Although most children (73%) received at least one intervention targeting developmental improvement, 40% of the rural living caregivers reported not receiving any type of intervention for their children, versus only 9% in urban communities (χ^2^(1) = 15.306, *p* < 0.001). Speech–language therapy was most often offered, followed by ABA.

### Satisfaction with services and support

Caregivers in urban and rural communities reported frequent unmet needs, due to unavailability of services or waiting lists, lack of information, and high costs. The majority reported frustration with getting services for their children. Approximately, one-third of the caregivers was very satisfied about the progress made by their children. There were no significant differences between urban and rural living caregivers (FQLS—satisfaction scale *U* = 1.930, *z* = –0.701, *p* = 0.48) (see Table 3).

**Table 3.** Caregivers’ satisfaction with services and support.

	Urban		Rural		Total sample		Chi-square (χ^2^) or Mann–Whitney *U*	*p* value
	41% (*n* = 54)		59% (*n* = 77)		*n* = 131			
	%	*n*	%	*n*	%	*n*		
*Unmet needs*								
Not eligible for services^a^	54%	28	68%	50	62%	78		
Services not available^b^	74%	39	84%	62	80%	101		
Waiting list^c^	58%	31	45%	34	50%	65		
Cost^d^	85%	46	72%	55	78%	101		
No information available^e^	67%	36	74%	56	71%	92		
Total unmet needs (median)	4.0		4.0		4.0		*U* = 2116	*0.76*
*Frustration in getting services* ^f^							χ^2^ = 2.012	*0.57*
Never	12%	6	9%	7	10%	13		
Sometimes	30%	15	24%	18	26%	33		
Usually	12%	6	8%	6	10%	12		
Always	46%	23	59%	44	54%	67		
*FQLS*—*satisfaction scale (median)*	2.0		2.0		2.0		*U* = 1930	*0.48*

Missing or “don’t know”: ^a^*n* = 5; ^b^*n* = 4; ^c^*n* = 2; ^d,e^*n* = 1; ^f^*n* = 6.

See Supplementary Table for FQLS—scale items scores.

### Factors associated with service usage and satisfaction

All independent variables included in the regression analysis were found to be linearly related to the logit of the dependent variable, as assessed via the Box–Tidwell procedure. Two outliers with standardized residual value >3 standard deviations were excluded from the analysis. The results of the logistic regression analyses are shown in Table 4. First, the predisposing factors were included (Model 1), showing that both younger age of the child and a higher maternal education, significantly predicted a higher chance of receiving any intervention for autism. When adding the enabling factors (Model 2), the area of residence significantly contributed to the prediction, with children in urban communities being much more likely to receive intervention than children in rural communities. Adding the need factors (Model 3) showed that the lower the child’s verbal level, the higher was the chance that intervention had been received. Also, the importance of support as perceived by caregivers significantly contributed to the prediction.

**Table 4.** Logistic regression predicting the likelihood of receiving any behavioural intervention for ASD for autism.

	Model 1 Nagelkerke *R*^2^*=.311*				Model 2 Nagelkerke *R*^2^*=.415*				Model 3 Nagelkerke *R*^2^*=.487*			*p*
	χ^2^(4)=27,74, *p<.001*				χ^2^(7)=38,73, *p<.001*				χ^2^(9)=47,13, *p<.001*			
	*B*	SE	Odds ratio (95% CI)	*p*	*B*	SE	Odds ratio (95% CI)	*p*	*B*	SE	Odds ratio (95% CI)	
predisposing factors												
*child's age*	-0.16	0.07	0.85 (0.75-0.97)*	**0.014**	-0.16	0.07	0.85 (0.74-0.98)*	**0.026**	-0.17	0.08	0.85 (0.73-0.99)*	**0.036**
*child's gender* ^a^	-1.04	0.63	0.35 (0.10-1.22)	0.100	-0.94	0.66	0.39 (0.11-1.42)	0.152	-1.33	0.72	0.27 (0.07-1.08)	0.065
*educational level mother*	0.67	0.21	1.96 (1.30-2.95)**	**0.001**	0.55	0.21	1.74 (1.15-2.64)**	**0.009**	0.74	0.25	2.09 (1.28-3.42)**	**0.003**
*educational level father*	0.04	0.17	1.04 (0.75-1.45)	0.812	0.05	0.18	1.05 (0.73-1.49)	0.802	0.03	0.2	1.03 (0.70-1.51)	0.888
enabling factors												
*Residence* ^b^					1.80	0.68	6.03 (1.59-22.94)**	**0.008**	2.5	0.81	12.20 (2.50-59.46)**	**0.002**
*Family financial problems* ^c^					0.56	0.63	1.74 (0.51-6.01)	0.379	0.52	0.66	1.69 (0.46-6.17)	0.431
*Stigma*					-0.30	0.37	0.74 (0.36-1.52)	0.413	-0.54	0.39	0.58 (0.27-1.25)	0.166
need factors												
*child's verbal level*									-0.54	4.86	0.59 (0.36-0.94)*	**0.027**
*Importance*									1.66	3.78	5.28 (0.99-28.28)*	**0.049**

SE: standard error; CI: confidence interval.

a Male = 1, female = 0.

b Urban = 1, rural = 0.

c Yes = 1, no = 0.

* *p* < 0.05; **p < 0.01.

Overall, satisfaction with services and the progression made by the child was not significantly predicted by the predisposing factors (Model 1), but introducing the enabling variables (financial problems and stigma) led to a statistically significant increase in explained variance of 18% (Δ*R*²). This model fitted the data best, since the addition of the need factors (Model 3) or intervention (Model 4) did not lead to a significant increase in the variation explained (Table 5).

**Table 5.** Hierarchical multiple regression predicting caregivers’ satisfaction with services and support.

	Module 1				Module 2				Module 3				Module 4			
	B	SE B	β	*p*	B	SE B	β	*p*	B	SE B	β	*p*	B	SE B	β	*p*
Predisposing factors																
*child's age*	0.01	0.02	0.08	0.407	0.01	0.02	0.06	0.532	0.01	0.02	0.06	0.558	0.01	0.02	0.08	0.421
*child's gender* ^a^	0.28	0.15	0.18	0.058	0.25	0.14	0.16	0.076	0.25	0.14	0.16	0.085	0.22	0.14	0.14	0.129
*educational level mother*	-0.04	0.05	-0.1	0.366	-0.06	0.05	-0.14	0.212	-0.06	0.05	-0.14	0.223	-0.07	0.05	-0.18	0.139
*educational level father*	0.04	0.04	0.11	0.33	0.02	0.04	0.05	0.65	0.02	0.04	0.05	0.666	0.02	0.04	0.04	0.683
Enabling factors																
*Residence* ^b^					-0.21	0.12	-0.16	0.087	-0.22	0.13	-0.16	0.099	-0.17	0.14	-0.13	0.217
*Family financial problems* ^c^					-0.34	0.15	**-0.22**	0.024*	-0.34	0.15	**-0.22**	0.025*	-0.35	0.15	**-0.23**	0.021*
*Stigma*					-0.16	0.08	**-0.2**	0.044*	-0.16	0.08	-0.19	0.051	-0.15	0.08	-0.18	0.077
Need factors																
*child's verbal level*									0.01	0.05	0.01	0.881	0.02	0.05	0.03	0.749
*Importance*									0.01	0.18	0.01	0.938	-0.01	0.18	-0.01	0.935
Use of services																
*Any intervention for ASD received* ^d^													0.17	0.15	0.12	0.266
R^2^	0.06				0.18				0.18				0.19			
ΔR^2^					0.12	*		**0.02**	0.00				0.01			

SE: standard error.

a Male = 1, female = 0.

b Urban = 1, rural = 0.

c Yes = 1, no = 0.

d Yes = 1, no = 0.

* *p* < 0.05.

## Discussion

This study represents the first systematic investigation of caregivers’ challenges and needs, and service use and satisfaction, as perceived by caregivers of autistic children in Morocco. It aims to identify support needs and to explore rural–urban disparities in Morocco, in service use and satisfaction. Urban and rural caregivers expressed similar priorities, that is, better health care services and education for their children, but reported different challenges in caring. Urban living caregivers expressed more concern about social-communicational deficits of their children, while rural living caregivers reported limited autonomy skills in their children as highly challenging.

Previous studies showed that limited skills, like dressing and toileting cause heavy burden for parents, and increasing daily living skills was found to decrease caregiver burden (Chaabène et al., 2018; Marsack-Topolewski et al., 2021). Better autonomy skills may also enlarge opportunities for inclusion. For instance, children who are not toilet trained may not be accepted in schools or experience social stigma. This may explain why daily living skills are more often a priority to rural living caregivers. In the absence of special needs support, children without sufficient autonomy skills may be excluded from education or social activities. Poor daily living skills, that is, lack of toilet training and poor meal-time behavior, were described as among the most important barriers to social integration for people with disabilities (Cocchiola & Redpath, 2017). A vicious circle may then arise: without access to school, families carry caregiving tasks all day without relief and lack support in autonomy skills training.

This study showed disparities in access to care. In accordance with Andersen’s model of health service utilization, the likelihood to receiving intervention was predicted by combined predisposing, enabling, and need factors. Residential area was an important factor, with 12 times higher odds of receiving intervention for children living in urban communities as compared to rural communities, despite similar verbal ability levels, age, and years since diagnosis. Furthermore, higher maternal education, younger age of the child, and lower verbal level contributed to the likelihood of receiving services. Possibly, the need of intervention is more obvious in young children with poor verbal skills. These results are in line with a study in Taiwan on autism care, where maternal education and urban residency, as well as perceived childcare pressure were influencing help-seeking behavior (Lung & Shu, 2022). In Australia, perceived caregivers’ needs predicted service use, but urban or rural residency did not contribute to the prediction (Willet et al., 2018). Country characteristics such as health care organization or (public) transport facilities may explain these differences.

Caregivers’ service satisfaction was best explained by the enabling factors “Financial problems” and “Stigma.” Since only a small proportion of the variance was explained by the model, other variables than the ones examined in our study are likely to play a role. Receiving intervention did not contribute to increased satisfaction and may imply limited quality of services offered. Another factor that may have impacted service dissatisfaction was that a significant proportion of autistic children in rural communities was not enrolled in school (40%), while in urban communities, 10% of the children did not attend school. Educational barriers are an important concern to caregivers of children with disabilities in MENA countries, particularly in rural regions (Alkhateeb et al., 2016; Hadidi & Al Khateeb, 2015). In Morocco, recent research showed that disability had no direct effect on access to education, but children with disabilities coming from poor families in rural areas were less likely to attend school (Trani et al., 2018).

### Implications and recommendations

These data point to significant challenges in fulfilling the promises enshrined in the United Nations “Convention on the Rights of Persons with Disabilities” and the “Sustainable Developmental Goals” for health care, inclusion, and education (United Nations, 2006; United Nations General Assembly, 2015). While there still is an urge to improve services and education in Morocco (Human Rights Watch, 2017), it is encouraging that inclusive education is a main goal of the Moroccan government (Ministry of Education, 2015). Our study shows that access to, and quality of inclusive education is among the top 3 priorities for caregivers, especially in rural areas. There are no public centers for autistic children In Morocco. Care or education is mostly provided by NGOs often founded by parents (sometimes, partially financially supported by the state) (Benmakhlouf et al., 2020). An autism program has recently been launched in Morocco, offering professional training in ABA interventions, and aiming to disseminate information to community-care professionals (Ministry of Solidarity, Women, Family and Social Development, 2019). Research has shown feasibility and effectiveness of autism interventions delivered by community-care workers in low-resource settings (Divan et al., 2019), which is promising for Morocco and other MENA countries. We recommend to examine efficacy of these interventions and the fit to local caregivers needs in Morocco.

One way to fit these interventions to local needs taking within-country urban–rural disparities in caregivers’ preferences into account, is to create modular programs. Modular programs allow for caregivers to choose topics that are most relevant to them. This empowers caregivers and facilitates shared decision-making, which is key to sustainable impact (Hyman et al., 2020). Based on our results, intervention modules addressing children’s autonomy skills may be valued more in rural communities, while intervention modules addressing social-communicational skills may be preferred by caregivers in urban areas. Furthermore, adaptive interventions may improve the fit between program content and individual needs. For instance, interventions may benefit from adapted content to meet living conditions (e.g. squat toilets vs seated toilets; sleeping with family members or sleeping alone), or to meet family conditions (e.g. extended families and child rearing; access to information).

Third, enabling factors should be addressed, because differences in perceived needs and service use are not only due to cultural disparities, but also due to practical issues such as costs, stigma, or access to information. In our sample, maternal education was among the predictors for intervention use. Understandable information in the spoken languages of a country and adapted to the cultural context is essential for caregivers to recognize developmental issues in their child, acknowledge the existence, and value of intervention and reduce stigma (Gordillo et al., 2020). Addressing enabling factors provide opportunity for policy-makers and program developers to reducing disparity in unmet caregiver’ needs (Karpur et al., 2019).

An essential question is how to effectively disseminate information to caregivers or community-care professionals in low-resource areas. The Internet is a rich tool, designated by urban living Moroccan caregivers as an important source of information. However, in rural communities, less than half of the caregivers used the Internet as an information source, probably as a result of limited Internet access, limited literacy levels, or a lack of culturally appropriate, understandable information for non-English-speaking caregivers. It is therefore highly important to adapt information to cultural contexts, local languages, and low-literate populations. Specific tools such as audio video tools or visual aids have successfully been used to communicate health information (Barclay & Bowers, 2017).

Finally, raising autism awareness among the general population and community-care professionals is highly important. Our data showed that children with higher verbal abilities were less likely to be receiving intervention. It is probable that autism symptoms in children with higher verbal abilities are less well recognized. When community-care professionals are well informed, they can play a crucial role in assisting caregivers to seek help (Lung & Shu, 2022).

### Strengths and limitations

The results of this study on Moroccan caregivers from urban and rural communities are based on convenience sampling. Although all children had been formally diagnosed according to parents, there was no diagnostic evaluation for this study. The lack of formal assessment of children’s developmental, adaptive and language level, is a limitation. However, to the best of our knowledge, there are no tests for language, developmental or adaptive functioning normed and validated in Morocco. A recent study showed that even assumed culture-free tests lead to significant bias in Morocco when applying norms from European or Eastern MENA countries (Lozano-Ruiz et al., 2021). We therefore used verbal ability level as a global indication of general functioning and social-communicative impairment. The validation of diagnostic measures in Morocco is urgently needed.

Caregivers were recruited via special schools or centers and parent associations, which means that we did not reach caregivers without access to these settings. Since the chances of getting a diagnosis are much lower in rural than in urban areas (Hsu et al., 2022; Lauritsen et al., 2014), the reported unmet needs are likely to be an underestimation. The actual percentage of autistic children not enrolled in school may be higher than in the present sample. In fact, only 42% of children with disabilities are enrolled in school in Morocco (Ministry of Solidarity, Women, Family and Social Development, 2015).

We took great effort to reach out to caregivers in different districts in Morocco and to include hard-to-reach families from rural communities. The importance for including these families in autism research in LMIC has been underlined by Divan et al. (2021). To ensure participation possibilities for caregivers with limited literacy skills, we interviewed the majority of participants in person. This carries, however, a risk for social desirability bias.

Although caregivers in the urban and rural group shared many characteristics, there were two important differences between groups that may have influenced the results. In rural communities, half of the participants were fathers, while in urban communities, mothers more often participated. Also, maternal educational levels were lower in rural communities than in urban communities. This may be one of the reasons why fathers more often responded to the invitation to participate, but cultural factors may also have played a role.

Finally, our sample included a broad age range. Future studies in larger groups are needed to investigate age-related caregivers’ needs in details. Despite these limitations, to our knowledge, this study represents the first systematic investigation of challenges and needs of caregivers of autistic children in Morocco and as such opens new avenues for future studies targeting implementation of services.

## Conclusion

The findings underline the importance of taking within-country urban–rural disparities in caregivers’ needs into account. Autistic children from rural communities were significantly less often attending school or receiving intervention than children from urban communities, despite similar age and verbal ability. Caregivers expressed similar needs for improved care and education, but different challenges in caring that may be driven by family factors, local conditions, or values. These differences have implications for public policy and program planning. Accessible and understandable information in local languages, both online and provided by community-care professionals, is needed to empower caregivers and increase service use. Adaptive or modular interventions reflective of regional needs, resources, and practices are important to improve shared decision-making and service satisfaction. Raising autism awareness and increasing knowledge, especially with respect to more able individuals with autism, as well as addressing enabling factors such as costs or stigma, may contribute to reducing disparities in health care utilization. In Morocco and other MENA countries, efforts to implementing autism programs are increasing. Additional research is needed to investigate the suitability of community-care autism programs in MENA communities, to empower autistic children and their caregivers.

## Supplemental Material

sj-docx-1-aut-10.1177_13623613221150086 – Supplemental material for Urban and rural differences in needs, service use and satisfaction among caregivers of autistic children in Morocco Click here for additional data file. Supplemental material, sj-docx-1-aut-10.1177_13623613221150086 for Urban and rural differences in needs, service use and satisfaction among caregivers of autistic children in Morocco by MV De Jonge, M Boutjdir, T El Korchi, H Torres, A Karpur, A Shih and A El Idrissi in Autism

## Notes

1. In accordance with Lord et al. (2022), we use “autism” as short for “autism spectrum disorder” and “autistic children” instead of children with autism because it is more acceptable to many autistic people.
