# Supplementary material for: Urban and rural differences in needs, service use and satisfaction among caregivers of autistic children in Morocco
Source: Autism. 2023 Feb 17;28(1):107–22. doi: 10.1177/13623613221150086 (PMC10771028; doi:10.1177/13623613221150086)
Supplement: sj-docx-1-aut-10.1177_13623613221150086 – Supplemental material for Urban and rural differences in needs, service use and satisfaction among caregivers of autistic children in Morocco [file sj-docx-1-aut-10.1177_13623613221150086.docx]

**Supplementary Table**

| ***ASS Stigma scale items*** |  |  |  |  |  |  |  |
| --- | --- | --- | --- | --- | --- | --- | --- |
| Feel helpless for having a child with autism. | |  |  |  |  |  |  |
| *Strongly disagree* | 19% | (10) | 22% | (17) | 21% | (27) |  |
| *Disagree* | 6% | (3) | 6% | (5) | 6% | (8) |  |
| *Agree* | 33% | (18) | 21% | (16) | 26% | (34) |  |
| *Strongly agree* | 37% | (20) | 42% | (32) | 40% | (52) |  |
| *Missing* | 6% | (3) | 9% | (7) | 8% | (10) |  |
| Worry if other people would know I have a child with autism. | | | |  |  |  |  |
| *Strongly disagree* | 50% | (27) | 45% | (35) | 47% | (62) |  |
| *Disagree* | 19% | (10) | 17% | (13) | 18% | (23) |  |
| *Agree* | 19% | (10) | 21% | (16) | 20% | (26) |  |
| *Strongly agree* | 9% | (5) | 12% | (9) | 11% | (14) |  |
| *Missing* | 4% | (2) | 5% | (4) | 5% | (6) |  |
| Other people would discriminate because of child with autism. | | | |  |  |  |  |
| *Strongly disagree* | 28% | (15) | 31% | (24) | 30% | (39) |  |
| *Disagree* | 22% | (12) | 16% | (12) | 18% | (24) |  |
| *Agree* | 43% | (23) | 23% | (18) | 31% | (41) |  |
| *Strongly agree* | 6% | (3) | 30% | (23) | 20% | (26) |  |
| *Missing* | 2% | (1) | 0% | (0) | 1% | (1) |  |
| Negative impact on me because of child with autism | | |  |  |  |  |  |
| *Strongly disagree* | 19% | (10) | 31% | (24) | 26% | (34) |  |
| *Disagree* | 19% | (10) | 19% | (15) | 19% | (25) |  |
| *Agree* | 35% | (19) | 27% | (21) | 31% | (40) |  |
| *Strongly agree* | 26% | (14) | 19% | (15) | 22% | (29) |  |
| *Missing* | 2% | (1) | 3% | (2) | 2% | (3) |  |
| ***FQLS-Importance scale items*** |  |  |  |  |  |  |  |
| Support for child to make progress in school | |  |  |  |  |  |  |
| *A little important* | 4% | (2) | 1% | (1) | 2% | (3) |  |
| *Important* | 17% | (9) | 3% | (2) | 8% | (11) |  |
| *Very important* | 80% | (43) | 88% | (68) | 85% | (111) |  |
| *Missing* | 0% | (0) | 8% | (6) | 5% | (6) |  |
| Support for child to make progress at home |  |  |  |  |  |  |  |
| *A little important* | 6% | (3) | 3% | (2) | 4% | (5) |  |
| *Important* | 20% | (11) | 6% | (5) | 12% | (16) |  |
| *Very important* | 72% | (39) | 91% | (70) | 83% | (109) |  |
| *Missing* | 2% | (1) | 0% | (0) | 1% | (1) |  |
| Support for child to make friends |  |  |  |  |  |  |  |
| *A little important* | 17% | (9) | 8% | (6) | 11% | (15) |  |
| *Important* | 20% | (11) | 13% | (10) | 16% | (21) |  |
| *Very important* | 63% | (34) | 77% | (59) | 71% | (93) |  |
| *Missing* | 0% | (0) | 3% | (2) | 2% | (2) |  |
| Good relationship with service providers |  |  |  |  |  |  |  |
| *A little important* | 7% | (4) | 3% | (2) | 5% | (6) |  |
| *Important* | 13% | (7) | 17% | (13) | 15% | (20) |  |
| *Very important* | 80% | (43) | 78% | (60) | 79% | (103) |  |
| *Missing* | 0% | (0) | 3% | (2) | 2% | (2) |  |
| ***FQLS-Satisfaction scale items*** |  |  |  |  |  |  |  |
| Support for child to make progress in school | |  |  |  |  |  |  |
| *Very dissatisfied* | 37% | (20) | 36% | (28) | 37% | (48) |  |
| *Neutral* | 19% | (10) | 27% | (21) | 24% | (31) |  |
| *Very satisfied* | 43% | (23) | 29% | (22) | 34% | (45) |  |
| *DK/missing* | 2% | (1) | 8% | (6) | 5% | (7) |  |
| Support for child to make progress at home |  |  |  |  |  |  |  |
| *Very dissatisfied* | 35% | (19) | 39% | (30) | 37% | (49) |  |
| *Neutral* | 37% | (20) | 30% | (23) | 33% | (43) |  |
| *Very satisfied* | 28% | (15) | 31% | (24) | 30% | (39) |  |
| *DK/missing* | 0% | (0) | 0% | (0) | 0% | (0) |  |
| Support for child to make friends |  |  |  |  |  |  |  |
| *Very dissatisfied* | 39% | (21) | 40% | (31) | 40% | (52) |  |
| *Neutral* | 35% | (19) | 29% | (22) | 31% | (41) |  |
| *Very satisfied* | 26% | (14) | 26% | (20) | 26% | (34) |  |
| *DK/missing* | 0% | (0) | 5% | (4) | 3% | (4) |  |
| Good relationship with service providers |  |  |  |  |  |  |  |
| *Very dissatisfied* | 24% | (13) | 31% | (24) | 28% | (37) |  |
| *Neutral* | 31% | (17) | 27% | (21) | 29% | (38) |  |
| *Very satisfied* | 44% | (24) | 38% | (29) | 40% | (53) |  |
| *DK/missing* | 0% | (0) | 4% | (3) | 2% | (3) |  |
